# Climate Variability Drives Dengue Transmission in Bangladesh

**DOI:** 10.3390/idr18030055

**Published:** 2026-06-09

**Authors:** Ayesha Siddiqa, Prosenjit Choudhury, Nabil Jahan Mahim, Suman Paul, Syed Sayeem Uddin Ahmed, Md Bashir Uddin

**Affiliations:** 1 Department of Epidemiology and Public Health, Sylhet Agricultural University, Sylhet 3100, Bangladesh; ayeshasiddiqa.vet@gmail.com (A.S.); c.prosenjit101@gmail.com (P.C.); pauls.eph@sau.ac.bd (S.P.); ahmedssu.eph@sau.ac.bd (S.S.U.A.); 2 Department of Medicine, Sylhet Agricultural University, Sylhet 3100, Bangladesh; mahiimnjm@gmail.com; 3 Faculty of Veterinary Medicine and Animal Science, Habiganj Agricultural University, Habiganj 3300, Bangladesh

**Keywords:** dengue, climate variability, generalized additive model (GAM), lagged effects, Bangladesh

## Abstract

Background: Dengue fever has emerged as a major public health concern in Bangladesh, with increasing incidence and geographic spread of outbreaks in recent years. This study aimed to investigate the lagged and non-linear associations between climatic factors and dengue incidence across all eight administrative divisions of Bangladesh from 2014 to 2025. Materials and Methods: An ecological time-series design was employed using monthly dengue case data (*n* = 741,338) and meteorological variables. A generalized additive model (GAM) with a negative binomial distribution was applied to account for overdispersion and capture complex relationships. Descriptive analysis was conducted to assess spatial heterogeneity, and choropleth maps were constructed to visualize the spatial distribution and regional variation in dengue burden across the country. Cross-correlation analysis was performed to identify significant lagged associations between climatic variables and dengue incidence. Results: Descriptive analysis showed substantial spatial heterogeneity, with the highest incidence observed in Dhaka (6.53 per 100,000) and the lowest in Sylhet (0.21 per 100,000). Choropleth maps illustrated distinct spatial distribution and regional variation in dengue burden across the country. Cross-correlation analysis identified significant lagged associations for temperature and rainfall (lag 1–3 months), humidity (lag 1–2 months), and wind speed (lag 2–3 months). The final GAM explained 88.6% of the deviance in dengue incidence (AIC = 7404.15; dispersion = 0.767). The approximate significance of smooth terms revealed that temperature at a lag of 1 month (*p* < 0.001, edf = 12.28), rainfall at a lag of 3 months (*p* < 0.001, edf = 2.85), and wind speed at a lag of 2 months (*p* < 0.001, edf = 2.25) were highly significant non-linear predictors of dengue transmission. Relative humidity was not significantly associated with dengue incidence. Non-linear effects revealed peak dengue risk at temperatures between 25 and 30 °C and moderate rainfall (~10 mm), particularly during monsoon months (June–October). A strong autoregressive effect indicated that prior dengue incidence significantly influenced current transmission. Conclusions: Overall, dengue transmission in Bangladesh is driven by complex, lagged, and non-linear interactions between climatic variables, seasonality, and regional factors. These findings provide critical evidence for climate-based early warning systems, enhance outbreak prediction, and inform evidence-based vector control strategies.

## 1. Introduction

Dengue is one of the most significant mosquito-borne viral diseases, affecting tropical and subtropical regions of the world, with an estimated 390 million cases of dengue infection and 96 million clinically apparent cases annually [1]. Dengue fever is caused by four distinct serotypes of the dengue virus (DENV 1–4), which belong to the family *Flaviviridae* and are transmitted primarily by the *Aedes aegypti*, a highly adaptable mosquito species [2].

In recent years, it has become a significant public health concern in Bangladesh, with a marked increase in the frequency and severity of outbreaks. The country has experienced several large-scale epidemics, most notably in 2019, 2023, 2024 and 2025. The 2019 outbreak reported 101,354 confirmed cases and 164 deaths, representing one of the largest outbreaks at the time. The 2023 epidemic was even more severe, with multiple reports indicating 321,179 cases and 1705 deaths within partial reporting periods, followed by 101,214 cases and 575 deaths in 2024, and 102,861 cases and 412 deaths in 2025, respectively, and it has been widely described as the largest and deadliest dengue outbreak recorded in the country [3,4,5,6]. These trends highlight the growing burden on the public health system. Dengue transmission in Bangladesh exhibits strong seasonality, with periodic epidemics exerting significant pressure on healthcare services [7]. Dengue risk has spread beyond traditional hotspots due to rapid urbanization, population growth, and changing environmental conditions, impacting both urban and peri-urban populations across several administrative divisions [8]. Transmission dynamics are shaped by a complex interaction of multiple factors, including circulating viral serotype/genotype, environmental conditions, and socio-demographic determinants, which influence vector biology, survival, and viral development [9].

Climatic variables such as temperature, rainfall, humidity, and wind speed are closely associated with dengue incidence. For instance, transmission peaks within a temperature range of approximately 26–29 °C, while temperatures around 28–32 °C can accelerate mosquito growth and shorten the extrinsic incubation period of the virus from about 12 to 7 days, but decline at extreme temperatures of 40 °C [9,10]. Rainfall contributes by increasing the availability of breeding sites, as water is essential for egg hatching and larval development, which typically takes about 7–10 days [11]. Relative humidity is an important determinant of mosquito survival and activity, with levels around 70–80% generally supporting a longer lifespan and more frequent blood-feeding, thereby increasing the potential for dengue transmission, particularly in high-risk and densely populated settings [12]. Wind speed may also influence transmission, as higher speeds can disrupt mosquito flight, whereas moderate conditions may facilitate short-distance dispersal and contribute to localized transmission [13]. Importantly, these effects are often delayed rather than immediate, reflecting the time required for mosquito maturation and viral incubation, and may vary across regions depending on local environmental and socio-demographic conditions.

Previous studies in South and Southeast Asia have consistently reported strong associations between climatic variability and dengue transmission. However, in Bangladesh, most research has been largely confined to Dhaka and has primarily relied on linear modeling approaches, such as generalized linear models, which may not fully capture the complex nature of the climate–dengue relationships. This represents an important gap, especially considering the diverse climatic and socio-environmental conditions across the country.

By applying this approach to division-level data across Bangladesh, this study aims to identify the lagged and nonlinear associations between key climatic factors and dengue incidence, and to examine how these relationships vary across different regions of the country. This analysis provides a more comprehensive understanding of climate–dengue dynamics beyond single-city perspectives, providing valuable insights for developing region-specific early warning systems and targeted vector control strategies. Ultimately, the findings are expected to support evidence-based public health planning and enhance preparedness for future dengue outbreaks in Bangladesh.

## 2. Materials and Methods

### 2.1. Study Design and Area

We conducted an ecological time-series analysis to investigate the association between climatic factors and dengue incidence across the eight administrative divisions of Bangladesh over 12 years (January 2014 to December 2025).

### 2.2. Data

Daily dengue case data were obtained from the Directorate General of Health Services (DGHS), Bangladesh, from January 2014 to December 2025. Data were aggregated to monthly counts at the division level. Aggregation to a monthly timescale was undertaken to mitigate excessive zero counts observed at finer temporal resolutions, which can adversely affect model stability and parameter estimation in count-based time-series analyses.

Daily meteorological data were obtained from the Visual Crossing Weather database for all divisions over the study period. Variables included mean temperature (°C), relative humidity (%), total rainfall (mm), and wind speed (km/h). All variables were aggregated to a monthly average to ensure alignment with dengue incidence data.

Division-specific monthly population data were estimated by linear interpolation between 2011 and 2022 census data. To project the population from July 2022 to December 2025, an exponential growth model was applied using division-specific annual growth rates provided by the Bangladesh Bureau of Statistics [14].

### 2.3. Statistical Formula for the GAM

The association between bioclimatic factors and Dengue incidence was modeled using a Generalized Additive Model (GAM) with a Negative Binomial (NB) distribution to account for overdispersion [15]. The model is specified as follows:

$$E\left(Dengue\_cases_{it}\right)=\;\mu_{it}$$

$$E\left(\mu_{it}\right)=\log\left(pop_{it}\right)+\beta_{0}+s_{1\left(\begin{array}{c} lag1\_de_{it} \end{array}\right)}+f_{\left(\begin{array}{c} temp_{lag1_{it}},\;\;\;Month_{t} \end{array}\right)}+s_{2\left(rain_{lag3_{it}}\right)}+s_{3\left(hum_{lag1_{it}}\right)}+s_{4\left(\begin{array}{c} wind\_lag2_{it} \end{array}\right)}+s_{5\left(tim{e\_index}_{t}\right)}+b_{i}$$

where *μ_it_* denotes the number of dengue cases for the *i*-th observation, and *log(pop_it_)* is the offset term used to account for the population at risk. *β*_0_ was the model intercept, *s*_1_ to *s*_4_ represents smoothing functions for the lagged bioclimatic and epidemiological predictors, *s*_5_*(time_index_t_)*: A smooth term capturing the long-term temporal trend (2014–2025), *f(temp_it_, Month_t_)*: A tensor product interaction *(te)* that captures the joint, non-linear effect of temperature and seasonality, and *b_i_* denote as a random effect term for the administrative divisions.

### 2.4. Statistical Analysis

All statistical analyses were conducted using R (version 4.4.2, R Foundation for Statistical Computing, Vienna, Austria). For generalized additive models (GAMs), we used the mgcv package. Statistical significance was defined as a two-sided *p*-value < 0.05.

Descriptive statistics were calculated for monthly dengue cases and climatic variables. Temporal trends and distributions of dengue were explored using time-series plots and box plots.

The lagged relationships between climatic factors and dengue were explored using cross-correlation functions (CCF). The lag times were determined based on correlation, significance, and biological plausibility. The lag times ranged from 0 to 3 months, reflecting the biologically plausible delays associated with the mosquito life cycle and viral incubation period.

The association of climatic variables with dengue was estimated by using a GAM with a negative binomial distribution. Non-linear associations were modeled using penalized regression splines. The lag effect of climatic variables on dengue incidence was considered up to 3 months, considering the life cycle of the vector.

The interaction effect of temperature and seasonality (month) was modeled using a smooth tensor product. Long-term trend effects were controlled by using a smooth function of time. Temporal autocorrelation effects were controlled by considering a one-month lag effect of dengue incidence.

The effect of division on dengue occurrence was considered by using a penalized spline with a random effect basis. The natural logarithm of population size was included as an offset variable to represent incidence rates. Non-climatic factors such as population density, socioeconomic status, and urban infrastructure were not explicitly included as covariates in the present model due to the unavailability of consistent, division-level time-series data. However, the inclusion of division-specific random effects partially captures underlying differences across regions that may arise from these unmeasured factors.

The correlation of climatic variables with each other was estimated by calculating Spearman’s correlation coefficient. Climatic variables with strong correlations (>0.7) were not included in multivariable analysis to avoid multicollinearity.

The best model was selected based on the Akaike Information Criterion. Model adequacy was checked by using standard diagnostic techniques.

## 3. Results

The descriptive statistics of dengue incidence and related climate factors of the eight divisions of Bangladesh from 2014 to 2025 are presented in Table 1. The total number of reported dengue cases was 741,338, with the highest number of dengue cases observed in Dhaka (*n* = 432,398), and the lowest number in Sylhet (*n* = 3383).

**Figure 1:**
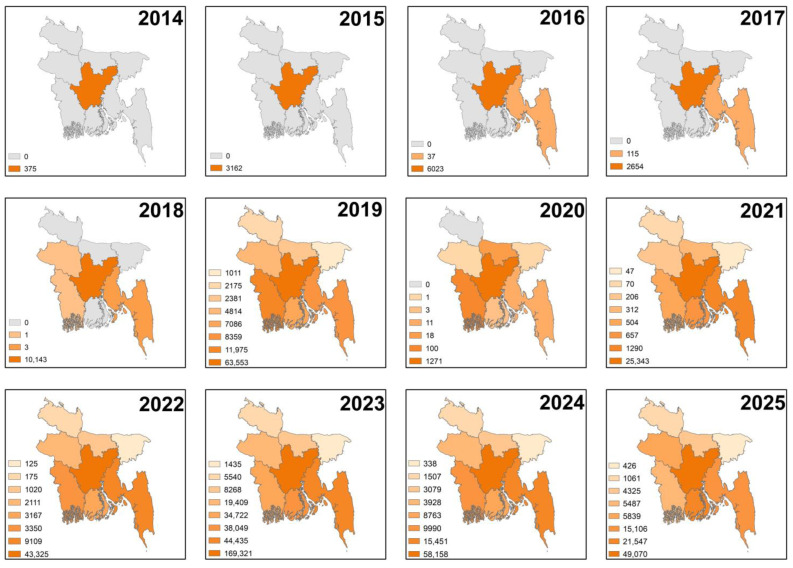
Spatiotemporal distribution of reported dengue cases in eight administrative divisions of Bangladesh during 2014–2025. The choropleth maps demonstrate high spatial heterogeneity and the gradual spread of dengue outbreaks over time.

The spatial distribution of dengue cases reported from all eight administrative divisions of Bangladesh between 2014 and 2025 is shown in Figure 1, showing clear geographic disparities and a gradual spread of dengue transmission to other regions in outbreak years. The overall incidence rate was 2.46 per 100,000 population, ranging from 0.21 in Sylhet to 6.53 in Dhaka. The overall mean temperature across all divisions was 25.76 ± 3.93 °C (range: 15.3–31.8 °C). Specifically, Dhaka and Chattogram exhibited the highest mean temperatures (26.7 ± 3.6 °C and 26.6 ± 3.1 °C, respectively), while Sylhet recorded the lowest (24.1 ± 3.9 °C). Similar variations were observed for maximum temperatures, with Khulna and Rajshahi showing the highest averages (31.3 ± 3.3 °C and 31.3 ± 3.9 °C) and Sylhet the lowest (29.7 ± 2.6 °C). For minimum temperatures, Chattogram and Dhaka recorded the highest averages (23.1 ± 3.9 °C and 23.1 ± 4.3 °C, respectively), whereas Sylhet showed the lowest (19.0 ± 5.1 °C).

The average relative humidity was 78.14 ± 5.65% (range: 52.9–91.7%), with Sylhet and Barisal reporting the highest average humidity (81.8 ± 5.3% and 81.8 ± 5.1%, respectively) and Dhaka the lowest (71.4 ± 7.1%). The average monthly rainfall ranged from 4.3 ± 4.2 mm in Rajshahi to 8.4 ± 8.9 mm in Sylhet, with an overall mean of 6.06 ± 5.86 mm (range: 0–44.1 mm). Wind speed varied widely between divisions, with the highest mean observed in Chattogram (21.7 ± 4.2 km/h) and the lowest in Sylhet (9.8 ± 2.6 km/h). The overall average wind speed was 13.82 ± 3.14 km/h (range: 5.5–32.5 km/h).

### 3.1. Explanatory Analysis

**Figure 2:**
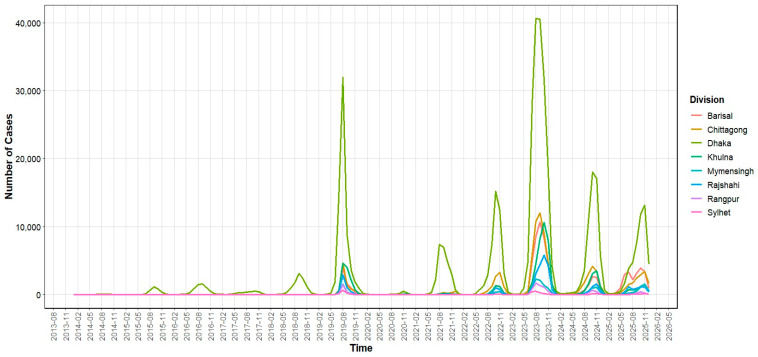
Division-wise dengue cases from January 2014 to December 2025.

Temporal variation in dengue incidence was observed throughout the study period (Figure 2). The highest number of cases was reported in 2023 with 321,179 cases, followed by 2025 with 102,861 cases. The lowest number of cases was reported in 2014 with 375 cases, followed by 1405 cases in 2020.

**Figure 3:**
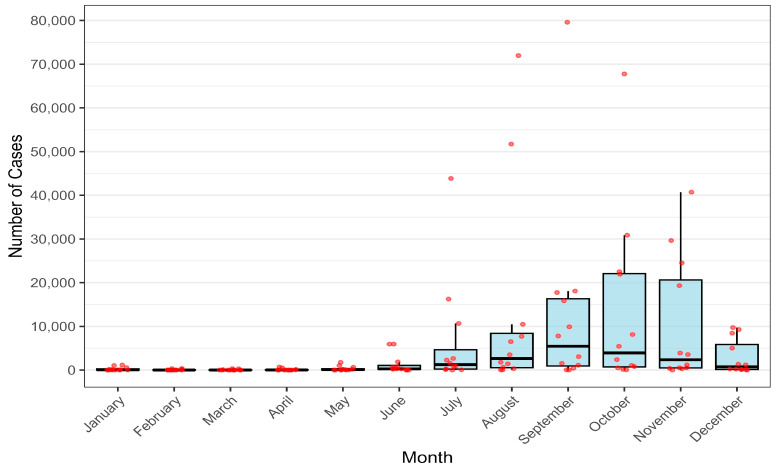
Distribution of dengue cases (monthly box plot). The blue boxes represent the interquartile range (IQR), and the dots represent outliers.

As shown in Figure 3, dengue cases remained consistently low from January to May. The peak period occurred between August and December, characterized by a substantial increase in both the mean number of cases and the frequency of major outbreaks (outliers) within the same epidemiological year.

### 3.2. Relationship Between Climatic Variables and Dengue Incidence

Cross-correlation analysis (Table 2) showed that several climatic parameters were significantly associated with dengue cases at various monthly lags. The most significant associations were observed for average temperature and rainfall at −1, −2 and −3 month lags, relative humidity at −1 and −2-month lags, and wind speed at −2- and −3 month lags.

The probability distribution of dengue incidence required for the GAM-NB model is highly over-dispersed (Mean = 643.5; standard deviation = 2879.137). The negative binomial distribution fitted the data well. We began by building GAMs to estimate the pattern of each influential variable in the incidence of dengue. Then, we used the Akaike Information Criterion (AIC) value to find the statistical performances of different models. The lowest AIC value yields the best-fit model.

Results from the best-fit GAM with a Negative Binomial distribution showed a significant association between dengue incidence and rainfall lagged 3 months, average temperature lagged 1 month, and wind speed lagged 2 months (lowest AIC = 7404.154; deviance explained = 88.6%, dispersion parameter = 0.767) (Table 3).

**Figure 4:**
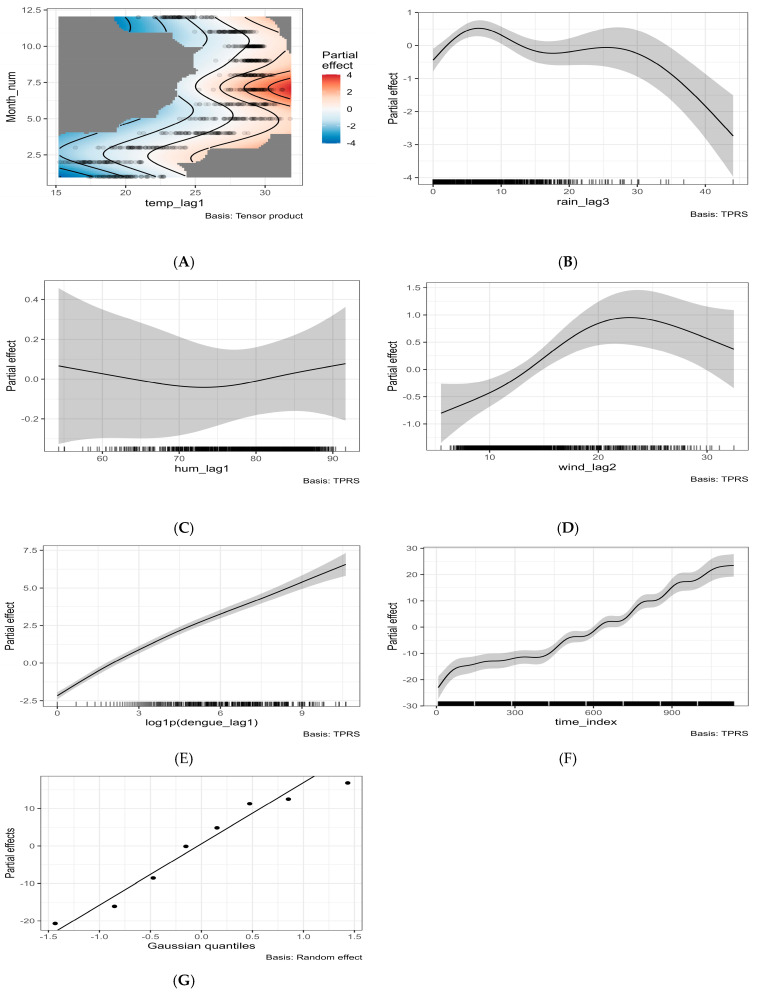
GAM-estimated partial effects of climatic and temporal predictors on dengue incidence (2014–2025). (**A**) temperature–seasonality interaction, (**B**) rainfall, (**C**) humidity, (**D**) wind speed, (**E**) lagged cases, (**F**) long-term trend, and (**G**) regional random effects. Y-axes show partial effects on the log scale; shaded areas represent 95% of Bayesian credible intervals. Rug marks along the x-axis indicate the distribution of observations.

The estimated effects of climate variables on dengue incidence are shown in Figure 4, which reveals different patterns. Most climate factors were statistically significant in a highly non-linear way. The interaction plot of temperature and month te(temp_lag1, Month_num) Figure 4A showed that during the epidemiological season, months are characterized by a non-linear smooth with a positive effect for months 6–10 (June to October), particularly when the temperature ranges between 25 °C and 30 °C (edf = 12.28). Average rainfall with 3 months lag s(rain_lag3) had a non-linear association with dengue incidence (edf = 2.85) (Figure 4B). A positive effect was seen for rainfall ranging between 0 and 15 mm, reaching a peak at 10 mm, and a negative effect was noted over 30 mm. Relative humidity with a 1-month lag s(hum_lag1) had no significant association with dengue incidence (Figure 4C). Average wind speed delayed 2 months s(wind_lag2) had a non-linear association and was characterized by an increasing effect for wind speed between 10 and 25 units, and a negative effect over 25 units (edf = 2.25) (Figure 4D). Log-transformed dengue incidence lagged 1 month s(log1p(dengue_lag1)) showed a significant linear positive effect on current incidence (Figure 4E). Time index s(time_index) also showed a general positive effect with non-linear wiggles over the study period (edf= 17.40) (Figure 4F).

## 4. Discussion

The study was conducted from 2014 to 2025 across the eight administrative divisions of Bangladesh to identify the relationship between dengue incidence and climatic factors and to examine how this relationship varies over time and across regions. We applied a GAM with a negative binomial distribution to better capture complex, non-linear patterns in dengue transmission over the 12 years. In addition, the GAM model incorporated smooth temporal trend terms and division-level random effects, which helped account for gradual changes in reporting intensity and underlying surveillance heterogeneity over time.

The model explained 88.6% of the variation in dengue cases, suggesting that climatic factors, along with temporal and regional effects, play an important role in dengue transmission. The inclusion of lag and interaction terms facilitated the identification of significant trends that are often missed in simpler linear approaches.

Our GAM analysis showed non-linear associations between dengue incidence and climatic factors, indicating both exposure–response relationships and delayed impacts over a 3-month lag period [16].

In this study, we found that temperature was positively associated with dengue incidence. An increase of 1 °C in the lag temperature resulted in a 78.2% significant increase in dengue risk. There was a rapid increase in dengue incidence when the temperature was between 28 °C and 32 °C, especially during the monsoon season [17,18]. This is supported by biological facts that show that higher temperatures hasten the development of the mosquito population and reduce the incubation period of the dengue virus [10]. However, it was also observed that higher temperatures (e.g., above 35 °C) resulted in a reduction in dengue transmission, probably because of the mortality of the mosquitoes at higher temperatures [10].

More importantly, the effect of temperature was significantly affected by seasonality. In fact, the highest risk was observed during the monsoon season when the favorable temperature is combined with humidity and the availability of breeding sites [19]. This finding highlights the importance of considering climatic variables collectively rather than in isolation [20].

In relation to rainfall, we observed a non-linear association between rainfall and dengue cases. Moderate rainfall could be favorable for mosquito breeding, while heavy rainfall was associated with a decreased risk of dengue. This could be attributed to the flushing effect of heavy rainfall, which interferes with mosquito breeding sites. Similar trends have been reported in other areas, where the effects of rainfall are time- and intensity-dependent [21,22]. A previous study in Thailand reported both positive and negative associations, depending on the areas [23]. Another study in Bangladesh also found that rainfall was negatively associated with dengue, but positively associated in some months [19].

Although relative humidity has been considered an important factor in dengue epidemiology, our study found no statistically significant association. Some studies reported that humidity increased disease incidence by improving mosquito survival [24,25]. Our findings are consistent with a study in Thailand that relative humidity was not associated with dengue transmission [26].

In this current study, wind speed was positively associated with the transmission of dengue, particularly with a lag effect. The non-linear effect suggests that moderate wind speed could facilitate the dissemination of mosquitoes, while higher wind speeds could interfere with mosquito flight and host-seeking behaviors, thereby reducing transmission [12,13,27,28].

The model also identified a strong autoregressive term (Lag 1), implying that the level of transmission currently is heavily predicated upon the level of cases the month prior. This “outbreak momentum” implies that when the critical level of community transmission is reached, the epidemic becomes self-sustaining, unaffected by minor climatic variability [29].

Dengue fever showed considerable geographical clustering between countries and within cities, owing to factors such as environment, climate, urban structure, and socioeconomic situations. Studies in Mexico, Brazil, and Bangladesh also reported similar results [30,31,32,33].

There was also evidence of spatiotemporal variation in the risk of dengue in various regions of Bangladesh. Some regions had higher baseline risk, which could be related to various factors, including urbanization, population density, healthcare facilities, and environmental factors. This variation likely reflects not only climatic differences but also underlying regional heterogeneity in demographic and socioeconomic conditions, which may influence dengue transmission dynamics across divisions. We also noticed a gradual increase in the overall incidence of dengue in Bangladesh over a period, suggesting a rising public health concern. This could be related to the rapid urbanization of Bangladesh, poor waste management, which provides perennial breeding sites, and improvements in disease surveillance systems. The occurrence of dengue in previously low-risk areas also supports the concept of an expanding endemic area [21].

While our analysis primarily focused on climatic drivers, it is important to acknowledge that substantial heterogeneity exists across Bangladesh’s administrative divisions in population density, socioeconomic status, and urban infrastructure. These non-climatic factors may act as important modifiers of dengue transmission by influencing vector breeding conditions, human–vector contact rates, and access to preventive measures [34,35]. In addition, factors such as vector control practices, sanitation conditions, healthcare accessibility, and improvements in surveillance systems may also play a crucial role in shaping dengue transmission dynamics across regions [36]. For example, densely populated and rapidly urbanizing regions such as Dhaka may experience higher transmission intensity due to increased human mobility and favorable breeding environments, whereas lower-incidence regions may differ in both environmental and social conditions [19,37]. The omission of these factors from the current model may partly explain the observed regional variation, and future studies should incorporate integrated climate–socioeconomic frameworks to provide a more comprehensive understanding of dengue dynamics.

While climatic, environmental, and socioeconomic factors contribute substantially to the observed spatial heterogeneity, viral dynamics may also play an important role in shaping dengue transmission patterns. Dengue virus circulates as four distinct serotypes (DENV-1 to DENV-4), and shifts in serotype dominance or the introduction of new genotypes can significantly influence transmission intensity and epidemic severity [38,39]. Differences in population immunity across regions, driven by prior exposure to specific serotypes, may result in varying levels of susceptibility and clinical incidence [40,41]. Although serotype-specific data were not available for inclusion in this analysis, these virological and immunological factors may partly explain the observed differences in dengue incidence between divisions such as Dhaka and Sylhet.

We acknowledge several limitations. Firstly, the quality of surveillance data may impact the outcome. In addition, potential changes in diagnostic practices and surveillance reporting over the study period may have influenced the long-term trends observed in dengue incidence. Secondly, individual-level factors like age, sex, and behavioral characteristics were not considered. Thirdly, the lack of entomological data limited our ability to evaluate mosquito dynamics. Additionally, the lack of serotype-specific and virological data restricted our ability to evaluate the role of viral dynamics and population immunity in shaping transmission patterns. Lastly, some data were excluded from the analysis because of the lag effect. Although minor autocorrelation remained, the overall findings were consistent and biologically plausible.

## 5. Conclusions

This study highlights the significant role of climatic factors in shaping dengue transmission dynamics in Bangladesh. Using a generalized additive model, we identified strong lagged and non-linear associations, with temperature emerging as the most influential predictor, particularly during the monsoon season. Rainfall and wind speed also showed complex effects, while humidity was not found to be significant. The model demonstrated high explanatory power, emphasizing the importance of temporal and regional variability. These findings stress the necessity for climate-informed early warning systems and targeted vector control strategies. Integrating climatic indicators into public health planning can enhance preparedness and help mitigate future dengue outbreaks in Bangladesh.

## Figures and Tables

**Table 1:** Descriptive statistics of dengue incidence and climate variables by division (2014–2025).

Division	Mean ± SD (Minimum–Maximum)					
	Dengue Cases	Incidence Rate	Temperature (°C)	Humidity (%)	Rainfall (mm)	Wind Speed (km/h)
Barisal	79,272	5.84	26.1 ± 4.0 (17.1, 31.0)	81.8 ± 5.1 (69.2, 90.4)	5.1 ± 5.0 (0.0, 23.5)	12.9 ± 3.0 (8.4, 20.2)
Chattogram	93,916	1.88	26.6 ± 3.1 (19.4, 30.5)	78.1 ± 6.1 (64.1, 89.8)	7.3 ± 8.7 (0.0, 36.8)	21.7 ± 4.2 (12.6, 32.5)
Dhaka	432,398	6.53	26.7 ± 3.6 (17.5, 31.2)	71.4 ± 7.1 (52.9, 85.8)	5.1 ± 4.6 (0.0, 19.3)	18.6 ± 4.8 (10.6, 31.1)
Khulna	66,129	2.56	26.4 ± 4.1 (17.2, 31.8)	80.5 ± 5.0 (69.1, 89.5)	4.7 ± 4.5 (0.0, 17.9)	12.9 ± 2.9 (8.2, 19.9)
Mymensingh	19,403	1.05	25.3 ± 4.1 (16.3, 30.1)	81.5 ± 4.7 (69.9, 87.9)	7.1 ± 6.5 (0.0, 21.8)	11.2 ± 2.4 (7.1, 16.8)
Rajshahi	36,309	1.2	25.8 ± 4.7 (15.3, 31.2)	78.3 ± 6.6 (55.0, 87.5)	4.3 ± 4.2 (0.0, 15.3)	11.9 ± 2.2 (8.1, 16.9)
Rangpur	10,528	0.4	25.1 ± 4.4 (15.4, 30.4)	79.7 ± 6.1 (59.2, 88.6)	6.5 ± 6.2 (0.0, 20.8)	11.6 ± 2.3 (7.7, 16.7)
Sylhet	3383	0.21	24.1 ± 3.9 (15.9, 29.5)	81.8 ± 5.3 (68.0, 91.7)	8.4 ± 8.9 (0.0, 44.1)	9.8 ± 2.6 (5.5, 21.9)

**Table 2:** Cross-correlation coefficients between bioclimatic variables and Dengue cases by Lag.

Lag (Month)	Temp (°C)	Humidity (%)	Rain (mm)	Wind (km/h)
0	0.183 *	0.233 *	0.121	−0.096
−1	0.29 *	0.31 *	0.28 *	0.093
−2	0.33 *	0.265 *	0.317 *	0.234 *
−3	0.32 *	0.128	0.247 *	0.322 *

* Significant at 0.05 level (*p* < 0.05).

**Table 3:** Model estimates of the effects of meteorological and temporal variables on Dengue incidence.

Smooth Terms	edf	Chi-Sq
s(log-lagged Dengue cases)	3.30	1122.19 ***
te(Temp_Lag 1, Month)	12.28	401.05 ***
s(Rainfall Lag 3)	2.85	32.63 ***
s(Humidity Lag 1)	0.56	0.72
s(Wind Speed Lag 2)	2.25	22.73 ***
s(Time Index)	17.46	527,785.63 ***
s(Division)	6.91	122.57 ***
Linear terms	Estimate	SE
Intercept	−15.36	5.12
Explained deviance	88.6%	
AIC	7404.15	

*** Significant at 0.001 level (*p* < 0.001). edf = effective degrees of freedom of the smooth function terms (edf > 1 indicates nonlinear relationships); SE = asymptotic standard error.

## Data Availability

All data generated or analyzed during this study are included in the manuscript.
